# Measurements of Tropospheric NO_2_ in Romania Using a Zenith-Sky Mobile DOAS System and Comparisons with Satellite Observations

**DOI:** 10.3390/s130303922

**Published:** 2013-03-20

**Authors:** Daniel-Eduard Constantin, Alexis Merlaud, Michel Van Roozendael, Mirela Voiculescu, Caroline Fayt, François Hendrick, Gaia Pinardi, Lucian Georgescu

**Affiliations:** 1 “Dunarea de Jos”, University of Galati, Faculty of Sciences and Environment, European Center of Excellence for the Environment, Str. Domneasca, Nr.111, Galati 800008, Romania; E-Mails: mirela.voiculescu@ugal.ro (M.V.); lucian.georgescu@ugal.ro (L.P.G.); 2 Belgian Institute for Space Aeronomy, Ringlaan-3-Avenue Circulaire B-1180, Brussels 1180, Belgium; E-Mails: alexis.merlaud@aeronomie.be (A.M.); michel.vanroozendael@aeronomie.be (M.V.R.); caroline.fayt@aeronomie.be (C.F.); francois.hendrick@aeronomie.be (F.H.); gaia.pinardi@aeronomie.be (G.P.)

**Keywords:** DOAS, spectrophotometer, nitrogen dioxide, mobile measurements

## Abstract

In this paper we present a new method for retrieving tropospheric NO_2_ Vertical Column Density (VCD) from zenith-sky Differential Optical Absorption Spectroscopy (DOAS) measurements using mobile observations. This method was used during three days in the summer of 2011 in Romania, being to our knowledge the first mobile DOAS measurements peformed in this country. The measurements were carried out over large and different areas using a mobile DOAS system installed in a car. We present here a step-by-step retrieval of tropospheric VCD using complementary observations from ground and space which take into account the stratospheric contribution, which is a step forward compared to other similar studies. The detailed error budget indicates that the typical uncertainty on the retrieved NO_2_tropospheric VCD is less than 25%. The resulting ground-based data set is compared to satellite measurements from the Ozone Monitoring Instrument (OMI) and the Global Ozone Monitoring Experiment-2 (GOME-2). For instance, on 18 July 2011, in an industrial area located at 47.03°N, 22.45°E, GOME-2 observes a tropospheric VCD value of (3.4 ± 1.9) × 10^15^ molec./cm^2^, while average mobile measurements in the same area give a value of (3.4 ± 0.7) × 10^15^ molec./cm^2^. On 22 August 2011, around Ploiesti city (44.99°N, 26.1°E), the tropospheric VCD observed by satellites is (3.3 ± 1.9) × 10^15^ molec./cm^2^ (GOME-2) and (3.2 ± 3.2) × 10^15^ molec./cm^2^ (OMI), while average mobile measurements give (3.8 ± 0.8) × 10^15^ molec./cm^2^. Average ground measurements over “clean areas”, on 18 July 2011, give (2.5 ± 0.6) × 10^15^ molec./cm^2^ while the satellite observes a value of (1.8 ± 1.3) × 10^15^ molec./cm^2^.

## 1. Introduction

Nitrogen dioxide (NO_2_) is an important trace gas in the photochemistry of Earth's atmosphere. In the stratosphere NO_2_ plays a key role as a catalyst of the ozone destruction [1]. In the troposphere NO_2_ is involved in the tropospheric ozone formation, having also a contribution to radiative forcing [2]. The main sources of NO_2_ are combustion of fossil fuels, biomass burning, lightning and microbiological processes in the soil [3]. Using remote sensing from space, the lifetime of tropospheric NO_2_ was estimated to be about 6 h in summer and 18–24 h in winter in mid-latitude industrialized regions [4].

Many studies show that exposure to high NO_2_ concentrations can create or aggravate respiratory or coronary diseases [5,6]. NO_2_ has seasonal variations which are both due to natural and anthropogenic causes [7].

The Differential Optical Absorption Spectroscopy (DOAS) technique [8,9] is a well established method that has been successfully applied to NO_2_ monitoring from ground and space. One of the first applications of DOAS was dealing with ground-based stratospheric and tropospheric NO_2_ measurements from zenith-scattered light observation [10,11]. Nowadays DOAS measurements are performed from a number of mobile platforms such as cars [12–15], balloons [16], ships [17], airplanes [18,19]. DOAS has also been used in satellite experiments such as SCIAMACHY [20], OMI [21] and GOME-2 [22] instruments, providing key observations to characterize NO_2_ at the global, regional and even at the urban scale [23].

Cars represent a cheap and accessible platform to perform mobile DOAS measurements. Several studies have been performed in different parts of the world, e.g., China [12], Mexic [13], USA [15], Germany [24], India [25], *etc.* These studies were focused on quantification of emissions of different trace gases by megacities (e.g., Delhi) or industrial plants.

In this paper we present the retrieval of tropospheric NO_2_ vertical column density (VCD) from zenith-sky mobile DOAS measurements using complementary observations from ground and space performed in Romania in the summer of 2011. In the next section the experiment and the methodology of tropospheric NO_2_ VCD retrieval are introduced. The results of mobile DOAS measurements, their error analysis, and comparison with satellite observations are then presented in Section 3, followed by Conclusions.

## 2. Methodology

### 2.1. Experimental and Instrumental Descriptions

**Figure 1.**
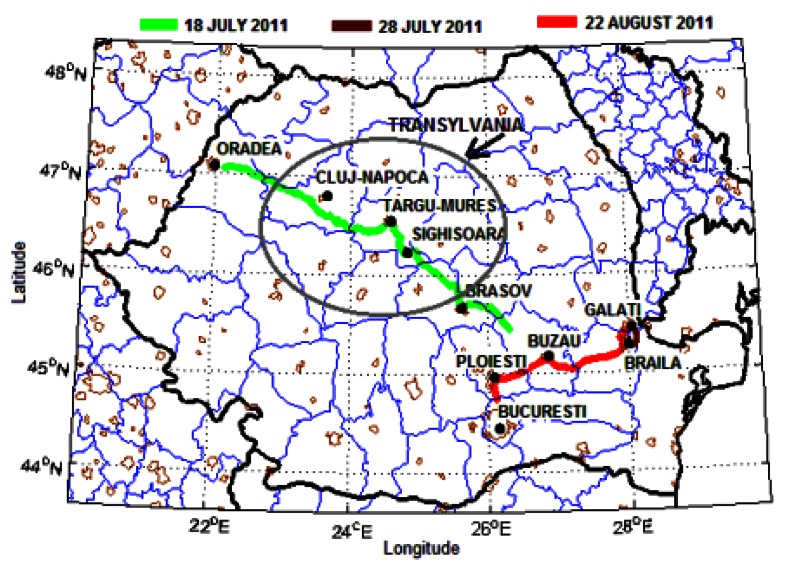
The tracks of mobile DOAS measurements performed in Romania.

Mobile DOAS zenith-sky measurements were performed in Romania during three days in summer 2011: July 18, July 28 and August 22. The measurements covered about 800 km in different regions of the country, including urban, rural and industrial areas (Figure 1). All measurements were performed under clear sky or mostly clear sky conditions. The time interval of measurements, the distance traveled and the main cities on road route are presented in Table 1.

Besides mobile measurements, a static DOAS experiment took place on 6 October 2011, at twilight-sunrise in a rural area close to Galati city.

**Figure 2.**
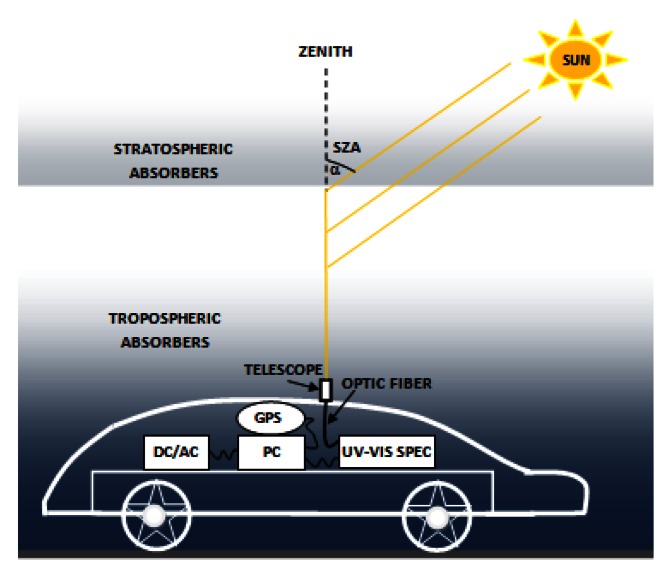
The mobile DOAS system.

The mobile DOAS instrument used in this work is based on a compact Czerny-Turner spectrometer (AvaSpec 2048 USB2, of 175 × 110 × 44 mm dimensions and 716 g weight) placed in a car. The spectral range of the spectrometer is 200–750 nm with 1.5 nm resolution (FWHM) with a focal length of 75 mm. The entry slit is 50 μm and the grating is 600 L/mm, blazed at 300 nm. The CCD detector is a Sony2048 linear array with a Deep-UV coating for signal enhancement below 350 nm. A flexible device (a piece of wood with a hole cached in a small metallic plate), mounted on the top of the road vehicle, holds the telescope achieving a 1.2° field-of-view with fused silica collimating lenses. The spectrometer is connected to the telescope through a 400 μm chrome plated brass optical fiber. Each spectrum is recorded by a laptop and georeferenced by a GPS receiver. The spectrometer and the GPS receiver are powered by the laptop USB ports. The entire set-up is powered by 12 V of the car through an inverter. Each measurement is a 5-second average of 10 scans accumulations at an integration time between 4–12 ms. The instrument was adapted from an instrument developed at BIRA-IASB [18]. Figure 2 presents the instrumental set-up.

### 2.2. Determination of Tropospheric Vertical Columns of NO_2_

The analysis of the zenith-sky spectra was performed using the QDOAS software [26], a program dedicated to the DOAS retrieval of atmospheric trace gases from ground-based and satellite measurements. The NO_2_ column density was retrieved in the spectral region 425–500 nm where NO_2_ has strong absorption lines. Absorption of O_3_, H_2_O and O_4_ can also be detected in the same region. The cross sections of NO_2_ at 298 K and 220 K [27], O_3_[28], O_4_ (http://www.aeronomie.be/spectrolab/o2.htm) and H_2_O [29] were included in the spectral fitting process. The “Filling-in” effect on Fraunhofer lines, also known as the Ring effect, originating from the rotational Raman scattering of molecular oxygen and nitrogen [30] was corrected by including into the fit a synthetic Ring spectrum calculated with QDOAS. Also a fifth degree polynomial representing the contribution of broad-band absorption in the atmosphere (Rayleigh and Mie scattering) was used in the DOAS analysis. The result of the DOAS fit is a Differential Slant Column Density (DSCD) of NO_2_ which is the difference between the slant column densities in the measured spectra (SCD) and in the Fraunhofer reference spectrum (SCD_ref_).

In our case, the tropospheric NO_2_ VCD retrieval from zenith-sky observations, or the mobile DOAS measurement results, involves complementary ground-based and satellite measurements. Determination of the NO_2_ amount in SCD_ref_ is required in order to determine the tropospheric NO_2_ VCD. Another important parameter is the air mass factor (AMF) which is needed to convert the resulted slant columns to vertical columns. The AMF is defined as the ratio between SCD and VCD (Equation (1)):

(1) $$\text{AMF}=\frac{\text{SCD}}{\text{VCD}}$$

The total slant column density in a measured spectrum (SCD_meas_) is defined by Equation (2):

(2) $$\text{SC}D_{\text{meas}}=\text{DSCD}+SCD_{\text{ref}}$$

where the NO_2_ content in the Fraunhofer reference spectrum or SCD reference (SCD_ref_) is unknown.

Stratospheric and tropospheric content of NO_2_ contribute to the measured slant column density, according to:

(3) $$AMF_{\text{tropo}}\times VCD_{\text{tropo}}+AMF_{\text{strato}}\times VCD_{\text{strato}}=\text{DSC}D_{\text{meas}}+SCD_{\text{ref}}$$

Thus the VCD of NO_2_ in the troposphere is given by:

(4) $$VCD_{\text{tropo}}=\frac{\text{DSC}D_{\text{meas}}+SCD_{\text{ref}}-AMF_{\text{strato}}\times VCD_{\text{strato}}}{AMF_{\text{tropo}}}$$

where AMF_strato_ and AMF_tropo_ are the stratospheric and tropospheric AMFs respectively.

#### 2.2.1. Deduction of SCD_ref_

A common way to quantify the SCD_ref_ is to use a Langley plot which is the graphical representation of DSCD at twilight *versus* the associated AMF [31]. By applying a linear fit to the resulting curve the slope gives the VCD and the intercept gives the SCD_ref_, as shown in Equation (5):

(5) $$\text{DSCD}=\text{VCD}\times \text{AMF}-SCD_{\text{ref}}$$

For NO_2_, due to the rapid variation of the NO_2_ concentration in the stratosphere at twilight, a photo-chemically modified Langley-plot is required [31–33]. We thus used scaled AMFs calculated using the radiative transfer model (RTM) UVspec/DISORT [34] coupled to the photochemical box-model PSCBOX [35–37]. Both box-model and RTM have been validated through several comparison exercises [36–39]. For the present study, the box-model PSCBOX is daily initialized with chemical and meteorological fields extracted from the 3-D chemical transport model (CTM) SLIMCAT [40] for the Jungfraujoch station (46.5°N, 8°E) in Switzerland which is similar in latitude to the locations of the mobile DOAS performed in Romania.

A spectrum with a low NO_2_ content recorded on 18 July 2011 at 8.82 UT and SZA = 33° was selected to represent and to determine the NO_2_ amount in the reference spectrum. The reference spectrum was selected after a pre-analysis of DSCDs with a random spectrum. The reference spectrum was recorded in a clean area in forested mountains, Transylvania (46.93°N, 22.83°E). The stratospheric contribution in this spectrum is supposed to be small considering the small SZA. All spectra presented in this paper have been analyzed with this spectrum. The benefits of using a single reference spectrum consist in avoiding different systematic errors which could affect the analysis process [41].

**Figure 3.**
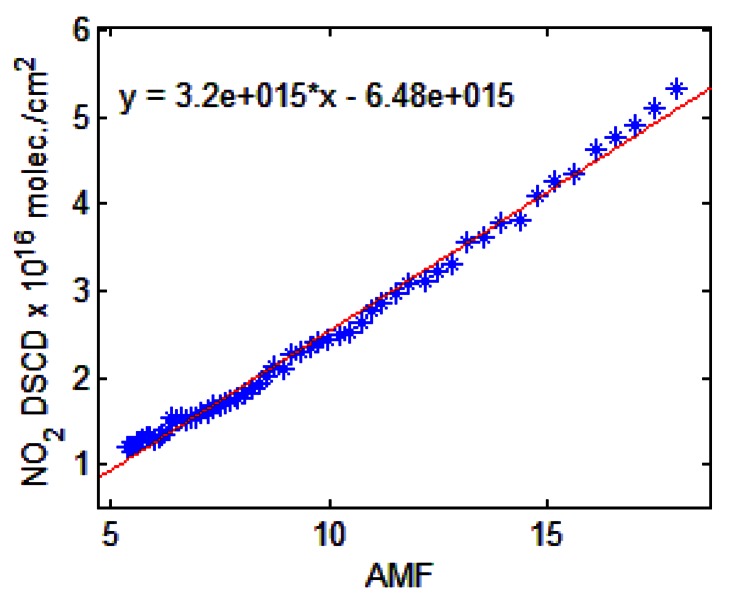
The Langley-plot for SZA 90°–80° of measurements on 6 October 2011 with respect to a reference spectrum measured on 18 July 2011.

The NO_2_ amount in the reference spectrum was obtained from ground-based zenith-sky measurements at sunrise on 6 October 2011. Figure 3 shows the photo-chemically corrected Langley plot for the SZA interval 90°–80°. The intercept of the corresponding straight line, representing SCD_ref_ and derived by linear least-squares regression, was found to be 6.48 ± 0.36 × 10^15^ molec./cm^2^.

#### 2.2.2. Deduction of VCD_strato_

Two methods for the determination of the stratospheric NO_2_ are described below. The first method is based on the derivation of stratospheric VCDs from measurements at sunrise by using the Langley-plot analysis presented in [42]. The authors used sunrise/sunset observations analyzed with a reference spectrum at 75°SZA (Equation (6)) which ensures that the potential impact of tropospheric NO_2_ variation in the Langley-plot analysis is reduced. This method can be successfully applied for sunrise/sunset observations performed in less polluted areas. Unfortunately it cannot be used for the retrieval of tropospheric NO_2_ from our mobile measurements because these were not performed at twilight, but during daytime. However, the above method was used to infer stratospheric content of NO_2_ using the static DOAS measurements on 6 October 2011. The AM variation of VCD_strato_ including the sunrise of 6 October 2011 will be estimated by extrapolating the slope resulted from Langley-plot to the simulation of the PSCBOX-model. The VCD_strato_ for 6 October 2011 was estimated at about 2.99 × 10^15^ molec./cm^2^ (6:18 UTC):

(6) $$VCD_{75{}^{\circ}\text{SZA}}=\text{DSCD}/(AMF_{\text{strato}}-AMF_{\text{strato}\_75{}^{\circ}\text{SZA}})$$

The second method to derive the stratospheric contribution is based on using the stratospheric NO_2_ columns obtained from the OMI instrument onboard AURA satellite. The assimilated vertical stratospheric columns were extracted from DOMINO (Dutch OMI NO_2_) Level2 product [43]. The stratospheric NO_2_ slant column from DOMINO is estimated by data-assimilation of OMI slant columns in TM4 chemistry-transport model. The selection of data satellite was made for an area with a radius with the half linear distance between the first and the last point of ground measurement. Variation of stratospheric NO_2_ in OMI data over Romania was found less than 5%. Table 2 lists the satellite overpass data sets that were used for the present analysis.

**Figure 4.**
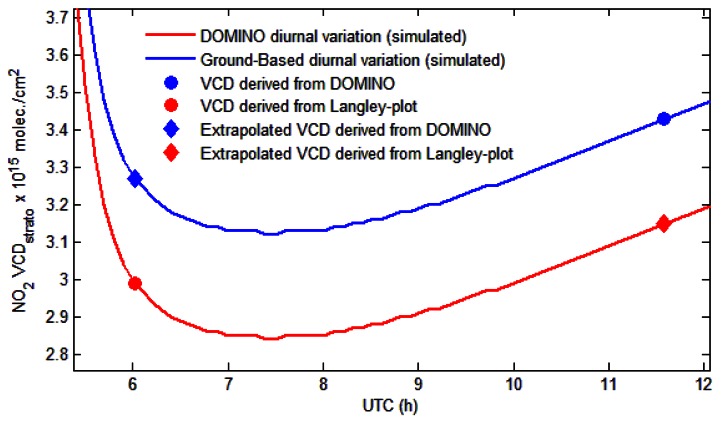
Comparison of the stratospheric NO_2_ between ground-based measurements and derived from OMI on 6 October 2011. The AM diurnal variation was simulated using the PSCBOX model.

Figure 4 compares the stratospheric VCD derived from the photo-chemically modified Langley-plot and from OMI. The diurnal variation of VCD_strato_ was calculated by extrapolating data from OMI measurements to the simulation of the PSCBOX model. Stratospheric NO_2_ columns from the DOMINO retrieval exceed the ground-based measurements by no more than 0.3 × 10^15^ molec./cm^2^. In a recent study [44], where DOMINO was compared with the SAOZ (Système d'Analyse par Observations Zénithal) instruments and the Network for the Detection of Atmospheric Composition Change (NDACC), a similar level of agreement was found.

Both methods presented in this chapter are suitable to retrieve the tropospheric NO_2_ content but the first method requires observations around twilight which are missing for the days with mobile measurements. Therefore, in this work, the DOMINO-based method was used to estimate stratospheric NO_2_.

#### 2.2.3. AMFs Simulations

**Figure 5.**
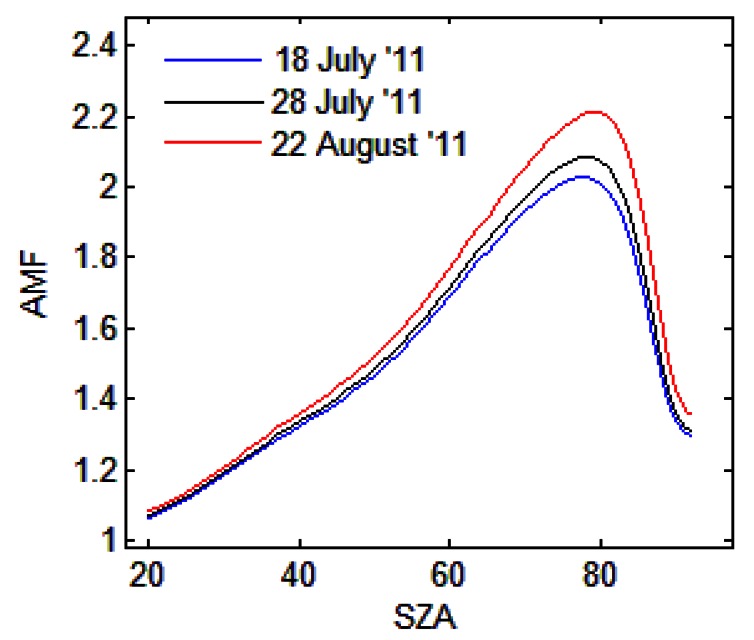
The tropospheric AMFs simulations for the three days of road measurements.

The AMFs simulations presented in this study were performed using the radiative transfer model (RTM) UVspec/DISORT. The RTM is based on the discrete ordinate method and deals with multiple scattering in a pseudo-spherical approximation. Given the wavelength, the observation's geometry relative to the Sun and the atmospheric state, the model calculates the scattered radiance and the absolute SCD of molecular absorbers. For the tropospheric AMF simulations we used NO_2_ profiles representative of Romania obtained from the CHIMERE model. The AMF_tropo_ simulations were made by setting a grid of 10 km altitude and the wavelength for NO_2_ simulation at 440 nm. Figure 5 shows the results of tropospheric AMFs simulations for the three days with road measurements in 2011.

## 3. Results and Discussions

**Figure 6.**
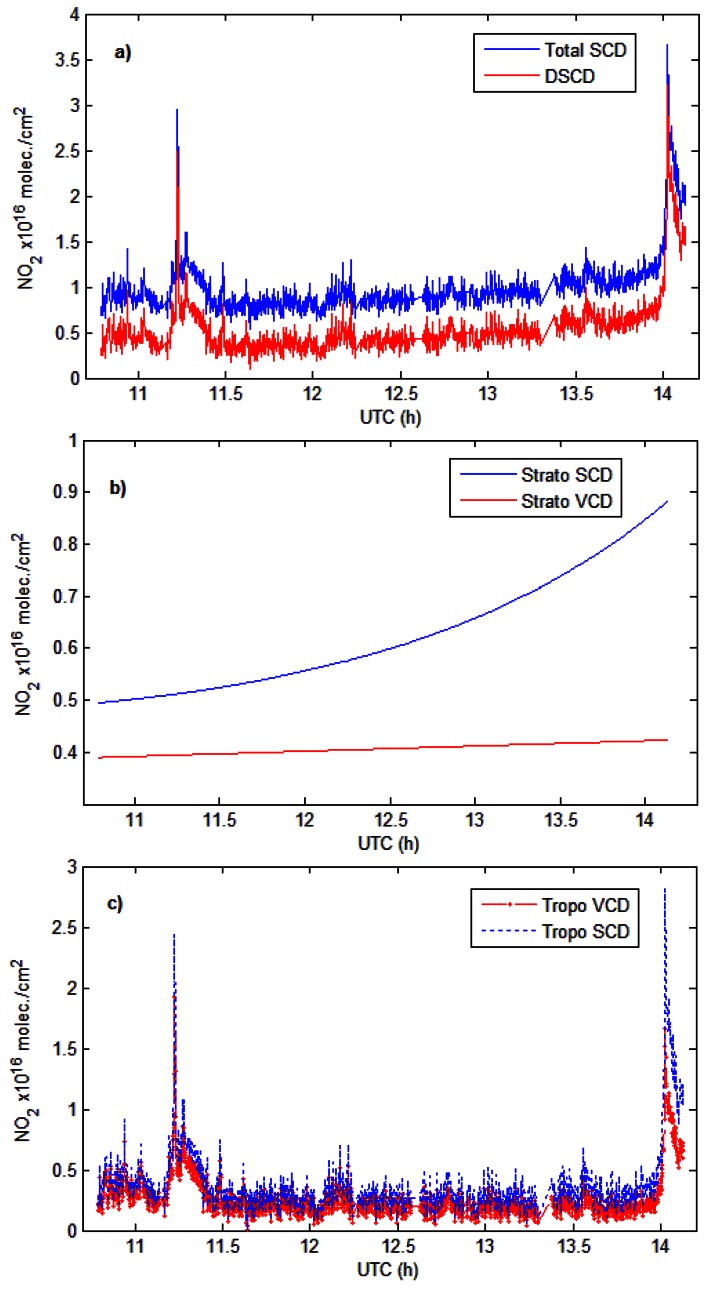
Time series of (**a**) DSCD and Total SCD; (**b**) Stratospheric SCD and VCD and; (**c**) Tropospheric SCD and VCD for 22 August 2011.

Figure 6 presents the spatial variation of tropospheric NO_2_ VCDs and the corresponding NO_2_ slant columns measured on 22 August 2011. The comparison of the DSCD with the VCD_tropo_ gives similar values around noon, which can be explained by the quasi vertical light path of solar photons through the atmosphere when the sun is high. Also this might indicate that a noon reference spectrum with a low NO_2_ content can correct for stratospheric slant column [42]. After noon, the DSCD increasingly and linearly deviates from the VCD_tropo_ at a rate of approximately 1% per degree of SZA. This is due the diurnal variation of stratospheric NO_2_.

### 3.1. Error Estimation

This section presents an analysis of the errors and of their propagation through the retrieval process. Each parameter used in the determination of tropospheric VCD has a contribution to the accuracy of the final retrieval. The error propagation on tropospheric VCD (σ_VCD_) can be expressed by Equation (7):

(7) $$\sigma_{{{\text{VCD}}^{2}}_{\text{tropo}}}={\left(\frac{\sigma_{\text{DSCD}}}{AMF_{\text{tropo}}}\right)}^{2}+{\left(\frac{\sigma_{SCD_{\text{ref}}}}{AMF_{\text{tropo}}}\right)}^{2}+{\left(\frac{\sigma_{SCD_{\text{strato}}}}{AMF_{\text{tropo}}}\right)}^{2}+{\left(\frac{SCD_{\text{strato}}}{AMF_{\text{tropo}}^{2}}**\sigma_{AMF_{\text{tropo}}}\right)}^{2}$$

Note that this expression assumes that the different error sources are uncorrelated, which is justified by the different origins of these error sources, which are described below. A correlation between the error of the DSCDs and the error of SCDref might exist if the NO_2_ cross section used in the DOAS fit is inaccurate, but this is not seen in our case. The error sources in our calculation are the following:

- The error on the DOAS fitting (σ_DSCD_) is calculated by QDOAS software and was found to be generally less than 1 × 10^15^ molec./cm^2^.
- The error on the estimation of the slant column in the reference spectra (SCD_ref_), which was obtained using a Langley plots (σ_SCDref_), depends on the one hand on the NO_2_ variation during twilight and on the other hand on the correct selection of the SZA interval for the AMF calculation used in the Langley plots. In our case, the error on the SCD_ref_ was calculated using the standard error formula for the case of simple linear regression.

The selection of an adequate SZA interval for the determination of SCD_ref_ is a critical issue. Table 3 shows that different SZA intervals used in the linear fitting may induce different values of intercepts. To rate the different possible choices, we consider the mean squared error (MSE) and the standard error of the linear regression. The interval with the smallest errors was selected as confidently representing the Langley plot. Using SZAs from 90° to 80°, the estimated σ_SCDref_ is of 3.56 × 10^14^ molec./cm^2^. As mentioned in Section 2.2.2, the measurements at large SZA are less contaminated by tropospheric NO_2_, On the other hand, when the sun is under the horizon, the available light is much reduced and the errors on the DOAS fit increase. The interval 90°–80° was chosen since it minimized the fit error.

- The error on stratospheric SCD (σ_SCDstrato_) is the uncertainty on the assimilated stratospheric slant column from DOMINO data product v2.0. This error is based on observation-forecast statistics [43–45] and is estimated to be 0.25 × 10^15^ molec./cm^2^.
- The AMF calculation can introduce important errors on the retrieval process of tropospheric VCDs. The tropospheric AMFs applied in this work were computed with DISORT using NO_2_ profiles from the CHIMERE chemistry transport model over Romania. In [46] the AMF_tropo_ uncertainties are estimated at 10%–20%, for SZA increasing from 20° to 85°. They use various input parameters for the radiative transfer simulation. Since their study presents an accurate description of AMF's uncertainties, their estimations of the error on tropospheric AMFs were used here.

After analyzing each error source we find that the major source of error comes from the DOAS fit. Our calculations show that, for all mobile measurements, the typical uncertainty on the retrieved NO_2_ tropospheric VCD is less than 25%.

**Figure 7.**
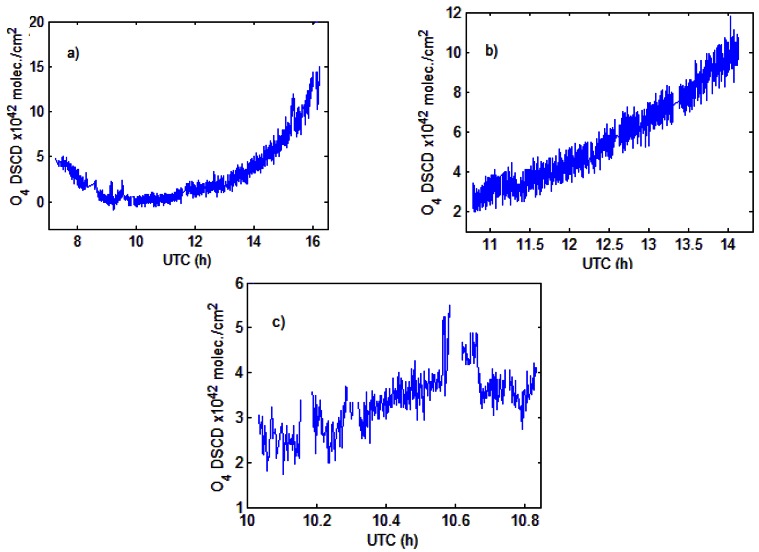
Diurnal variations of O_4_ DSCDs absorptions for clear sky or mostly clear sky conditions on (**a**) 18 July 2011; (**b**) 28 July 2011 and (**c**) 22 August 2011.

As an additional selection criterion, O_4_ absorption measurements performed together with NO_2_ were used as an indicator for the variation of the radiative transport through the atmosphere [47]. In a clear sky atmosphere, the oxygen collisional dimer O_4_ absorption varies with the square of the oxygen pressure [48] and is weakly dependent on temperature [49]. Analyzing the O_4_ DSCDs resulting from the DOAS fit (Figure 7) the time variations of O_4_ can therefore give an idea of the light path enhancement through the atmosphere.

### 3.2. Comparison of Our Mobile Measurements with Satellite Data

**Figure 8.**
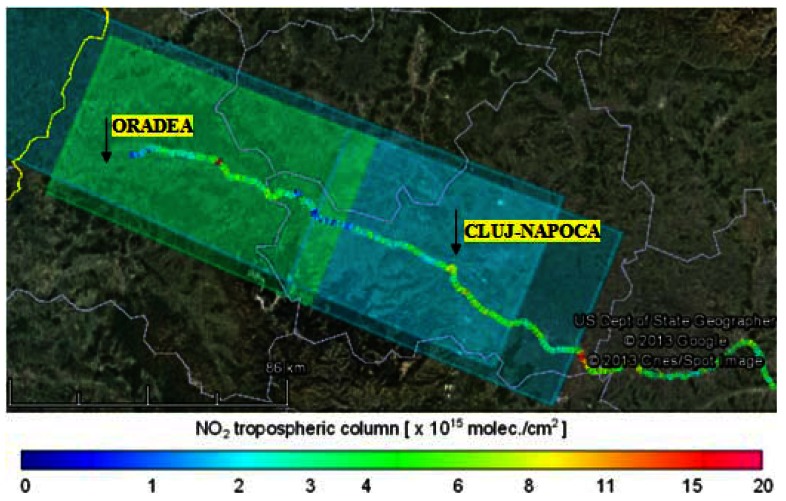
Color coded tropospheric NO_2_ VCD for the Oradea-Cluj Napoca road measurements on 18 July 2011 (line) and GOME-2 tropospheric NO_2_ VCDs for the same day (rectangles).

In this section the tropospheric VCDs retrieved from mobile zenith-sky measurements are compared with the tropospheric VCDs derived from the nadir measurements of OMI and GOME-2 instruments, onboard the AURA and Metop-A satellites. Only pixels with the geographic center located below 20 km distance of mobile measurements for GOME-2 and respective 10 km for OMI were selected for comparison. The largest number of mobile measurements were made on 18 July 2011, but they could not be used for comparison with OMI observations because the satellite passed over Romania close to the West border of the country, while the ground measurements were performed inside the country, more than 50 km away from the closest satellite pixel. The only day when matching observations from both satellites exist was 22 August 2011. In Figure 8 a color coded comparison between road measurements and GOME-2 observations made on 18 July 2011 is shown.

The next figure presents measurements of GOME-2, with a horizontal resolution of 40 × 80 km^2^, mobile observations and the average of mobile observations according to pixel size of GOME-2. Satellite observations and averages of mobile measurements are accompanied by error bars. The background NO_2_ satellite loading was considered to be the minimum observed by satellite, which is (1.8 ± 1.3) × 10^15^ molec/cm^2^.

The NO_2_ background for mobile measurements was obtained by averaging the measurements between 22.8 and 23.1 longitude, where NO_2_ is low and relatively constant. This gives (2.5 ± 0.6) × 10^15^ molec./cm^2^ which is close to the minimum observed by satellite.

**Figure 9.**
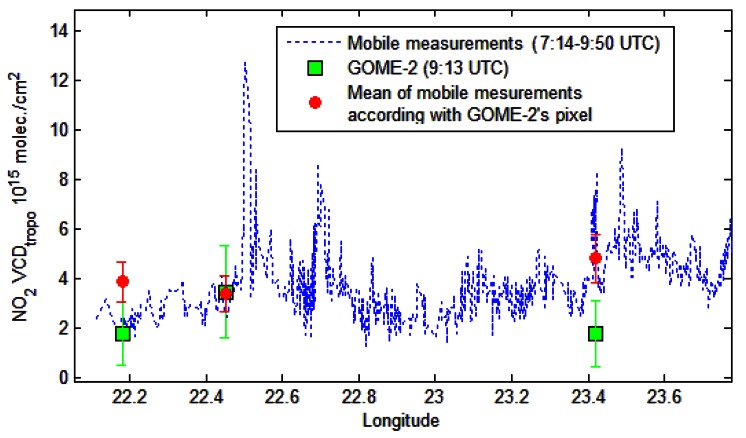
Comparison of tropospheric NO_2_ VCD obtained from mobile DOAS zenith-sky measurements with GOME-2 observations (18 July 2011) and corresponding error bars.

After averaging the tropospheric NO_2_ columns along the spatial extent of the satellite pixel, for a location centered on 47.03°N, 22.45°E which is influenced by emissions of two important cities, Oradea (47.05°N, 21.94°E) and Cluj-Napoca (46.76°N, 23.60°E), the NO_2_ loadings were found to be similar. For this location the averaged mobile measurements amount to (3.4 ± 0.7) × 10^15^ molec./cm^2^ while GOME-2 observations indicate (3.4 ± 1.9) × 10^15^ molec./cm^2^. Figure 9 shows that satellite measurements in the pixels located at 22.2 and 23.4 longitude are significantly smaller than mobile measurements. The satellite cannot “see” NO_2_ emissions from very small areas (e.g., NO_2_ located around to the road with low traffic, small cities or villages) while mobile measurements can determine the NO_2_ at a very small resolution comparing to GOME-2 observations (40 × 80 km^2^). The area of measurements is generally “clean” of NO_2_ being covered mostly by forested mountains. We believe that an increased number of measurements inside the GOME-2 pixels, away from point sources of NO_2_, would reduce discrepancies after averaging the mobile measurements according to the size of the satellite pixel.

**Figure 10.**
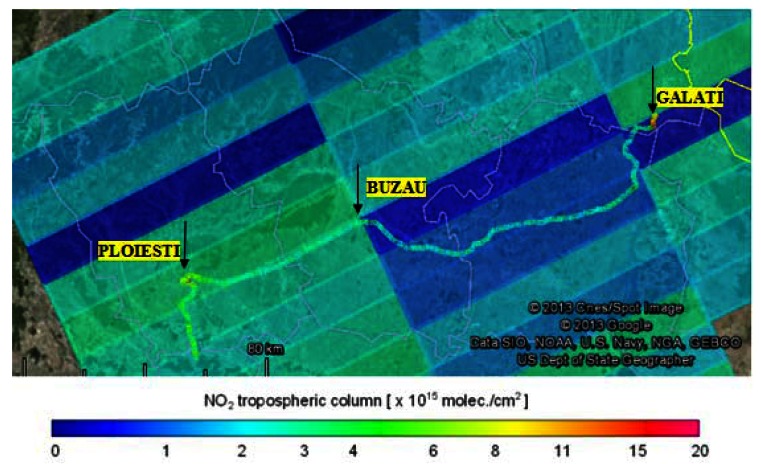
Color coded tropospheric NO_2_ VCD for the Ploiesti - Galati road measurements (line) on 22 August 2011 and OMI tropospheric NO2 VCDs (rectangles) for the same day.

In Figure 10 we present the color coded comparison between road measurements and OMI measurements for another day, 22 August 2011.

**Figure 11.**
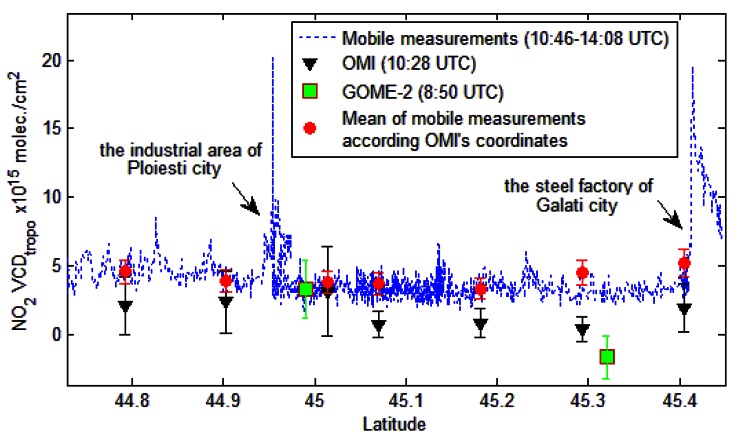
Comparison of tropospheric NO_2_ VCD deduced from mobile DOAS zenith-sky measurements with OMI and GOME-2 observations (22 August 2011) and the corresponding error bars.

Figure 11 shows the tropospheric NO_2_ VCD derived from zenith-sky measurements corresponding to OMI and GOME-2 satellite observations, as a function of latitude. The first NO_2_ peak of mobile measurements was recorded close to a power plant around Ploiesti city and the second one was recorded around a steel and iron factory which is close to the Galati city. Between these cities no other strong source for NO_2_ emissions exists along the trajectory of the mobile measurements. The NO_2_ background for mobile measurements is estimated at about (3.5 ± 0.6) × 10^15^ molec./cm^2^. The satellite instruments generally underestimate NO_2_ content, especially in less polluted areas. However, the difference between satellite and mobile observations are reduced in areas where strong NO_2_ sources are encountered.

The smoothing effect is significant even for OMI measurements with the finest resolution (13 × 24 km^2^) for areas with localized NO_2_ sources. For a better comparison we average the ground measurements according to the size of the OMI pixels. This reduces the differences between the two sets of measurements. For instance, at 11:10 UTC, around coordinates 44.9°N, 26.1°E, which corresponds to the industrial area of Ploiesti city, GOME-2 reports a NO_2_ content of (3.3 ± 2.1) × 10^15^ molec./cm^2^, OMI gives (3.2 ± 3.2) × 10^15^ molec./cm^2^ and the average of mobile measurements shows (3.8 ± 0.8) × 10^15^ molec./cm^2^.

**Figure 12.**
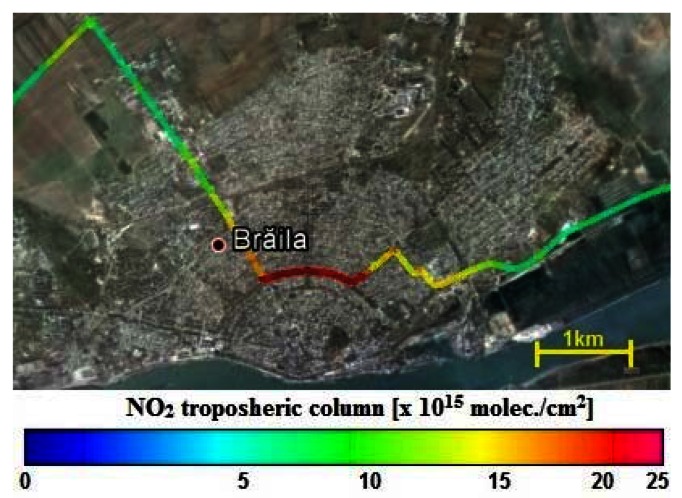
The NO_2_ “city center effect” in Braila town figured with mobile zenith-sky DOAS measurement on 28 July 2011.

Figure 12 shows a “zoom” inside of an OMI pixel made by mobile DOAS measurements in Braila city on 28 July 2011. Braila city is a relatively small city (77.9 km^2^), whose main source of atmospheric pollution is due to local transportation. The maximum mobile tropospheric NO_2_ column recorded is (2.5 ± 0.5) × 10^16^ molec./cm^2^ and its corresponding average for an OMI pixel is (4.9 ± 1.2) × 10^15^ molec./cm^2^. For the same area, OMI reports a column of about (2.4 ± 1.3) × 10^15^ molec./cm^2^. The significant difference between OMI and mobile DOAS measurements is probably caused by the smoothing effect inside the pixel. The total surface of an OMI pixel is 312 km^2^ which is 4 times larger than the area where local transportation increases the NO_2_ content. The size of the satellite pixel and the magnitude of the hot-spot are most likely responsible for these discrepancies.

## 4. Conclusions

Zenith-sky mobile DOAS measurements were performed in Romania over large areas during three days in summer 2011. These were complemented by static measurements at twilight. A detailed method for retrieval of tropospheric NO_2_ VCDs using ground-based and satellite observations is presented. This method presents three steps. First the NO_2_ amount in the reference spectrum is determined from ground-based measurements performed at twilight-sunset, using a photo-chemically modified Langley plot. The second step consists in determining the stratospheric NO_2_ SCD content by means of the assimilated vertical stratospheric column from the satellite DOMINO NO_2_ product. By comparing stratospheric NO_2_ VCDs derived from DOMINO with twilight observations for the morning of 6 October 2011, it is found that the stratospheric NO_2_ from DOMINO exceeds the ground-based measurements by no more than 0.3 × 10^15^ molec./cm^2^. To determine the diurnal variation of stratospheric NO_2_, PSCBOX model simulations were used. Finally, the tropospheric VCD was determined using a tropospheric AMF calculated with the RTM UVspec/DISORT, using NO_2_ profiles representative of Romania obtained from the CHIMERE model. Error propagation on tropospheric VCD was estimated to be less than 25%.

The comparison of the mobile DOAS measurements with results of OMI and GOME-2 instruments shows that satellite-based measurements generally underestimate ground based mobile observations for areas with VCD NO_2_ background smaller than 5 × 10^15^ molec./cm^2^. In contrast, the difference between mobile observations and satellite measurements is reduced in areas with strong NO_2_ emissions. Averaging mobile measurements in order to better match the horizontal extent of satellite pixels nearby a NO_2_ pollution source on 18 July 2011 gives (3.4 ± 0.7) × 10^15^ molec./cm^2^, while the corresponding result of GOME-2 for the same area gives shows (3.4 ± 1.9) × 10^15^ molec./cm^2^. On 22 August 2011, around Ploiesti city (44.99°N, 26.1°E), GOME-2 measures a NO_2_ content of (3.3 ± 1.9) × 10^15^ molec./cm^2^, OMI gives (3.2 ± 3.2) × 10^15^ molec./cm^2^ while the corresponding average of mobile measurements is (3.8 ± 0.8) × 10^15^ molec./cm^2^. Over “clean areas” with forested mountains, the average of ground measurements on 18 July 2011 gives (2.5 ± 0.6) × 10^15^ molec./cm^2^ while the satellite observations show (1.8 ± 1.3) × 10^15^ molec./cm^2^.

One of the possible future applications for mobile measurements such as presented in this work would be to derive more accurate NO_2_ emissions data around point sources. The comparison of mobile measurements with simultaneous Multi-Axis (MAX-) DOAS measurements and satellite observations will be the subject of a future paper.

## Figures and Tables

**Table 1.** Coordinates and temporal coverage of the three experiments.

**Day**	**Time Interval UT**	**Distance Traveled**	**Main Cities on Road Route**
18 July 2011	7.23–16.22 h	500 km	Oradea (47.05°N, 21.94°E)
			Cluj-Napoca (46.76°N, 23.60°E)
			Targu Mures (46.54°N, 24.55°E)
			Sighisoara (47.05°N, 21.94°E)
			Brasov (45.65°N, 25.60°E)

28 July 2011	9.99–10.84 h	30 km	Galati (45.43°N, 28.03°E)
			Braila (45.26°N, 27.95°E)

22 August 2011	10.78–14.12 h	250 km	Ploiesti (44.94°N, 26.03°E)
			Buzau (45.15°N, 26.81°E)
			Braila (45.26°N, 27.95°E)
			Galati (45.43°N, 28.03°E)

**Table 2.** Information on the OMI data used in this work.

**Day**	**Orbite Nr.**	**Overpass Time**	**Stratospheric VCD**
18 July 2011	37,628	11:35 UT	4.49 × 10^15^ molec./cm^2^
28 July 2011	37,778	10:34 UT	3.96 × 10^15^ molec./cm^2^
22 August 2011	37,413	12:05 UT	4.03 × 10^15^ molec./cm^2^
6 October 2011	38,433	11:35 UT	3.39 × 10^15^ molec./cm^2^

**Table 3.** Variation of intercept and errors resulted from Langley-plots.

**SZA**	92°–80°	91.5°–80°	90°–80°	91.5°–75°	91.5°–72°	90°–75°
**Intercept (×10^15^)**	−9.98	−8.51	−6.48	−5.77	−5.07	−4.31
**MSE (×10^15^)**	2.36	1.51	0.89	1.74	1.75	1.10
**σ_SCDref_(×10^14^)**	7.13	4.80	3.56	3.75	7.13	5.09
